# Effect of Different *Arnica montana* L. Plant Parts on the Essential Oil Composition, Antimicrobial Activity, and Synergistic Interactions with Antibiotics

**DOI:** 10.3390/molecules30183812

**Published:** 2025-09-19

**Authors:** Piotr Sugier, Danuta Sugier, Małgorzata Miazga-Karska, Aleksandra Nurzyńska, Beata Król, Łukasz Sęczyk, Radosław Kowalski

**Affiliations:** 1 Department of Botany, Mycology and Ecology, Institute of Biological Sciences, Maria Curie-Skłodowska University, 20-033 Lublin, Poland; piotr.sugier@mail.umcs.pl; 2 Department of Industrial and Medicinal Plants, University of Life Sciences in Lublin, 20-950 Lublin, Poland; beata.krol@up.lublin.pl (B.K.); lukasz.seczyk@up.lublin.pl (Ł.S.); 3 Chair and Department of Biochemistry and Biotechnology, Medical University of Lublin, 20-093 Lublin, Poland; malgorzata.miazga-karska@umlub.edu.pl (M.M.-K.); aleksandra.nurzynska@umlub.edu.pl (A.N.); 4 Department of Analysis and Evaluation of Food Quality, University of Life Sciences in Lublin, 20-704 Lublin, Poland; radoslaw.kowalski@up.lublin.pl

**Keywords:** *Arnicae anthodium*, *Arnicae rhizome*, *Arnicae radix*, essential oil, antibacterial activity, EO-antibiotic synergy

## Abstract

*Arnica montana* L. (mountain arnica) is a medicinal plant with diverse biological activities commonly used in pharmacy and cosmetics. The attributes of *A. montana* are related to e.g., the concentration and chemical composition of its essential oils (EOs). Therefore, the objective of this study was to: (i) characterize the chemical composition of EOs obtained from mountain arnica flower heads, rhizomes, and roots used as a pharmacopoeial material, (ii) demonstrate the effects of particular EO types and their combinations on antibacterial activity, and (iii) demonstrate the effect of the presence of *A. montana* EOs and their combinations with commercial antibiotics on their antibacterial activity. Essential oils obtained by hydrodistillation from different parts of *A. montana* were screened for their chemical composition and antibacterial properties. The chemical composition of the EOs was determined using the GC–MS technique. E-caryophyllene, caryophyllene oxide, germacrene D, farnesyl acetate, and dodecanal were the main components of the EO distilled from the flower heads. In turn, 2,5-dimethoxy-p-cymene, 2,6-diisopropylanisole, p-methoxyheptanophenone, and thymol methyl ether were the main molecules detected in the EO from the *A. montana* rhizomes and roots. The data clearly indicate that the presence of mountain arnica EO alone and in the interaction with commercial antibiotics (amoxicillin, ciprofloxacin, metronidazole) has a beneficial effect on their antibacterial activity.

## 1. Introduction

*Arnica montana* L. (mountain arnica) is endemic to Europe and is protected in most European countries [1]. Tinctures or ointments containing extracts of this plant have been used for several centuries as part of the treatment of various ailments, including contusion, wounds, and rheumatism [2,3,4,5,6,7]. *A. montana* is a source of sesquiterpene lactones, flavonoids, terpenoids, phenolic acids, and essential oils (EOs) [8,9]. These secondary metabolites are characterized by antioxidant, anti-inflammatory, antiseptic, antiradical, antisclerotic, and anticancer effects and a wide range of diverse biological activities [4,5,6,10,11,12,13,14,15,16,17,18]. While the antioxidant and antibacterial activities of arnica flower heads (*Arnicae anthodium*) have been extensively studied [19], the underground parts (*Arnicae rhizome* and *Arnicae radix*) have been investigated less comprehensively. Previous studies determined the chemical composition of EO derived from rhizomes and roots, with 2.5 dimethoxy-p-cymene as the main component; additionally, a very high EO yield: 4.05% from the rhizomes and 1.89% from the roots of two-year old plants was reported. Other studies have also demonstrated the chemical composition of EO derived from rhizomes and roots, the absence of EO toxicity at the concentrations used in the experiment, and its anti-cancer properties [20]. The plant part (rhizome, root) and plant age have been indicated as the determinants of the composition of EO and, consequently, its biological activity. However, the antibacterial properties of arnica EO have not been investigated to date, and the knowledge of the biological properties of compounds contained in the underground parts of the plant is still insufficient.

Previous studies determined the composition of EO from different parts of arnica plants (mainly flower heads) growing in different soil and climatic conditions, in different European countries, in different periods, and under the impact of different experimental factors [6,11,13,16,20]. Simultaneous analyses of the chemical composition and biological activities of EOs obtained from different parts of the same plants allow elimination of the influence of genetic variation and such factors as soil conditions, weather conditions, and agrotechnical conditions on the EO content and composition, providing insight into the phytochemical potential of this species and, consequently, its biological potential. On the other hand, acquisition of EO from different plant parts maximizes the utilization of herbal raw materials with diverse chemical compositions. In literature, there are many examples of essential oils obtained from different plant parts with different EO chemical compositions and diverse antioxidant and antibacterial activities, as in the case of *Nepeta persica* [21], *Leonurus japonicus* [22], and *Taxodium distichum* [23]. In turn, combining EOs diversifies the chemical composition and creates the potential for synergism between EO components, resulting in distinct biological activity. Therefore, in our research, we expected different reactions of individual bacterial strains to different chemical compositions of EO. Moreover, significant qualitative differences were demonstrated between EOs from aboveground and underground parts, which had not been previously analyzed in one mountain arnica plant population.

Many research reports have shown additivity or moderate synergism between EOs and antibiotics, indicating that EOs may offer possibilities for reducing antibiotic use, which addresses the WHO warnings related to the threat of bacterial antibiotic resistance [24,25,26,27,28,29], even at notably reduced concentrations [30]. This approach is one of the strategies for combating antibiotic-resistant bacteria in the future [31,32]. Moreover, the use of combined EOs and in conjunction with antibiotics or a dietary supplement shows their new biological functions and new ways and possibilities of application in medicine, pharmacy, and various branches of industry [33]. The present study clearly fits into this research trend. Mountain arnica EO has not been analyzed so far in the context of enhancing the action of antibiotics; therefore, this is a completely novel approach. The aim of this study was to: (i) characterize the chemical composition of EOs obtained from *A. montana* flower heads, rhizomes, and roots used as a pharmacopoeial material; (ii) demonstrate the effects of particular EO types and their combinations on antibacterial activity, and (iii) demonstrate the effect of the presence of *A. montana* essential oils and their combinations with commercial antibiotics on their antibacterial activity.

## 2. Results and Discussion

### 2.1. Chemical Characteristics of Essential Oils

The chemical composition of the essential oils obtained from the mountain arnica flower heads (FH), rhizomes (RH), and roots (RO) is presented in Table 1. The results of the analysis showed qualitative differences in the chemical composition of the studied EO distilled from the individual parts of the plants. Fifty-three components in the essential oil obtained from the flower heads (FH) constituted approx. 97.49%, 24 components in the essential oil obtained from the rhizomes (RH) accounted for approx. 97.51%, and 21 components in the essential oil obtained from the roots (RO) constituted approx. 96.74% of the total EO content. The FH essential oil was dominated by sesquiterpene hydrocarbons (55.43%), oxygenated aliphatic hydrocarbons (14.97%), and monoterpene hydrocarbons (10.41%). E-caryophyllene, caryophyllene oxide, germacrene D, farnesyl acetate, dodecanal, and decanal were the main ingredients in the arnica flower heads. The underground part had a completely different chemical composition from that in the flower heads. Fourteen EO components were common in the FH and RH essential oils, and 11 ingredients were common in FH and RO. The RH and RO essential oils were dominated by aromatic phenyl compounds and ketones, which accounted for 83.77% and 83.00%, respectively. The chemical composition of the essential oils from the rhizomes and roots was similar, with 21 common components in both EOs. 2,5-dimethoxy-ρ-cymene, 2,6-diisopropylanisole, p-methoxyheptanophenone, thymol methyl ether, and α-isocomene were the main components in the RH and RO essential oils.

### 2.2. Differentiation of Essential Oils

Research has shown that different plant parts differ in their chemical composition and exhibit different biological activities [35,36,37,38,39]. The results of investigations of *A. montana* EO presented in the literature were obtained in various experiments conducted at different times, in different geographic regions, and in different climatic and soil conditions; most often, researchers investigated *Arnicae anthodium* [6,12,16,40,41]. For the first time, we demonstrate the significant variation in the composition of EOs extracted from different plant organs of the same plants and, in consequence, their different biological activity. E-caryophyllene, caryophyllene oxide, germacrene D, farnesyl acetate, dodecanal, and decanal were the main ingredients in the FH essential oil (Table 1). The last three molecules mentioned were not found in the RH and RO essential oils. In turn, 2,5-dimethoxy-ρ-cymene, 2.6-diisopropylanisole, p-methoxyheptanophenone, thymol methyl ether, and α-isocomene were the main components in the RH and RO EOs but not in the FH material.

The present study showed that the essential oils distilled from the flower heads were characterized by a totally different chemical composition from that in the EO from the underground part, whereas the rhizomes and roots were chemically similar. The total number of components (over 50) and the contribution of monoterpenes and sesquiterpenes are consistent with other studies on the mountain arnica species conducted in different regions of Europe [6,12,16,40]. However, the composition of the dominant components is slightly different. As reported by Vidic et al. [41], EOs obtained from *A. montana* flower heads were mainly composed of *n*-Hexadecanoic acid (16.1%), *n*-Tetradecanoic acid (10.5), decanoic acid (5.0%), *n*-Tricosane (4.2%), and dodecanoic acid (3.4%). In turn, Judžentienė and Būdienė [12] found that (Z, Z)-geranyl linalool (14.7%), (2E, 6E)-farnesol (4.6%), *n*-Eicosane (4.5%), (Z, E)-geranyl linalool (3.2%), and docosane (3.0%) were the main components of EO obtained from flower heads. The present results agree with data presented by Ristić et al. [40]. The researchers showed that E-caryophyllene (31.5–34.6%), germacrene D (12.5–16.3%), trans-α-ionone (3.9–4.3%), and decanal (2.7–5.3%) were the main components, depending on the date of harvesting the flower heads. In turn, in our previous studies, E-caryophyllene (11.1–18.0%), farnesyl acetate (13.5–16.1%), decanal (6.7–10.4%), germacrene D (5.8–8.9%), and caryophyllene oxide (4.5–6.7%) were the main ingredients, and the EO composition depended on the maturity of flower heads [16]. However, an experiment on the impact of foliar boron fertilization on the essential oil content and yield of components from flower heads showed modification of the chemical composition of EO with caryophyllene oxide (12.9–18.2%), E-caryophyllene (11.6–15.2%), germacrene D (5.1–8.8%), dodecanal (4.6–5.5%), cumene (4.5–5.7%), and decanal (2.3–5.5%) as the main components [6]. Generally, the variation in the chemical composition of EO obtained from flower heads results from a number of factors, such as the genotype of cultivated plants, soil conditions, climatic conditions, plant age, development phase, and agrotechnical factors used in field experiments [6,12,13,16,20,40,41,42], which influence the production and composition of secondary metabolites.

In the present study, the main components in the EOs from the arnica RH and RO were 2,5-dimethoxy-ρ-cymene (49.8%, 52.2%, respectively), 2,6-diisopropylanisole (19.3%, 20.8%), p-methoxyheptanophenone (14.6%, 12.4%), thymol methyl ether (6.5%, 1.5%), and α-isocomene (1.0%, 2.2%). In a study conducted by Pljevljakušić et al. [13], 33 components were identified in EO distilled from rhizomes and roots of 3-year-old plants, and this number was substantially higher than that reported in the present study. The EO of mountain arnica plants growing in the subalpine climate in Serbia contained 2,5-dimethoxy-p-cymene as the main component. However, the content of this component was substantially lower and amounted to approx. 30% in EO obtained from rhizomes and approx. 40% in EO obtained from roots, compared to the data presented in this paper. As in our study, the next main constituents of essential oils obtained from underground plant parts were aromatic compounds: thymol methyl ether, p-methoxyheptanophenone, and 2,6-diisopropylanisole, and the content of these components was in the range of 9.6–10.6%, 7.0–7.5%, and 12.8–14.1%, respectively. In contrast, the chemical composition of the *A. montana* EOs analyzed in this study differs largely in terms of the number of EO components from the data reported by Danila et al. [42] The authors showed that, among 35 compounds identified in EOs from *A. montana* rhizomes and roots in populations originating from the East Carpathians, the major constituents were 3-t-butyl-1,2-dimethoxybenzene, tetradecane, thymol, non-3-en-2-one, and thymol methyl ether. It can therefore be concluded that the chemical composition of EO from plants cultivated in the climatic conditions of Poland and Serbia differs significantly from the chemical composition of oils obtained from their natural habitats in the West Carpathians [42].

A previous study of the chemical characteristics and anticancer activity of mountain arnica essential oil from rhizomes and roots showed 2,5-dimethoxy-ρ-cymene as a definitely dominant component [20]. The percent share of this ingredient and 2,6-diisopropylanisole in EO obtained from rhizomes and roots was similar to the data presented in the current study. However, there were differences in the content of the other two main EO components: p-methoxyheptanophenone and thymol methyl ether. The content of p-methoxyheptanophenone in the EO from rhizomes and roots of four-year-old *A. montana* plants analyzed in the previous experiment was 6.2% and 5.1%, respectively [20], but it was over two-fold higher in the present study. In turn, the content of thymol methyl ether in the EO from the underground organs was definitely lower.

### 2.3. Antibacterial Activity of Essential Oils

**Figure 1:**
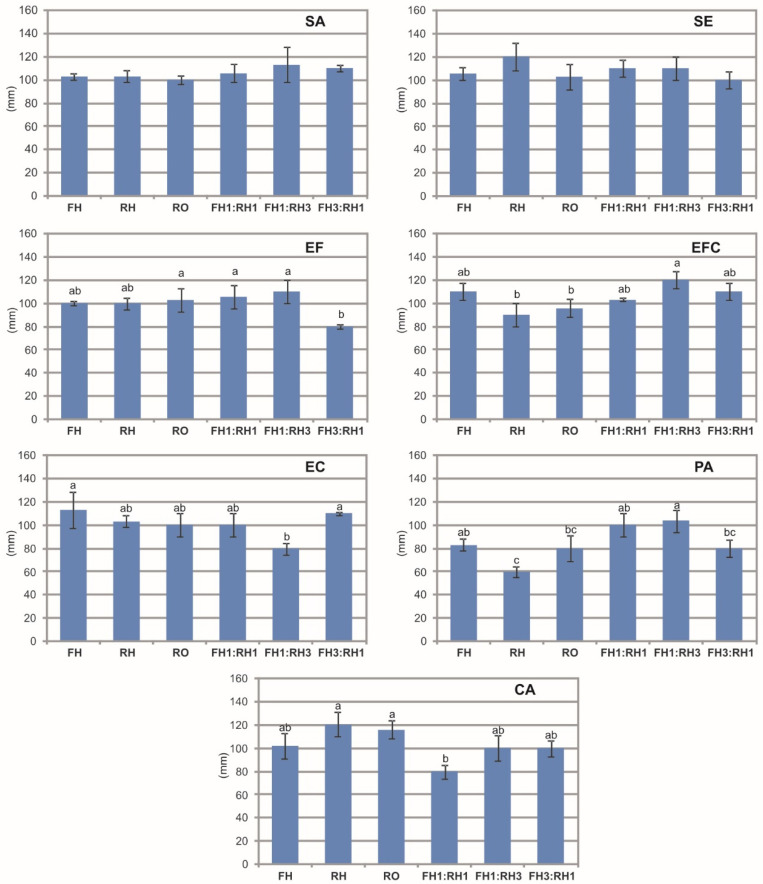
Zones of bacterial growth inhibition induced by the *A. montana* essential oils and their combinations. SA—*S. aureus* ATCC 25923, SE—*S. epidermidis* ATCC 12228, EF—*E. faecalis* PCM 896, EFC—*E. faecalis* (w.c.i.), EC—*E. coli* ATTC 25992, PA—*P. aeruginosa* ATCC 27853, CA—*C. acnes* ATCC 11827; FH—essential oil obtained from flower heads, RH—essential oil obtained from rhizomes, RO—essential oil obtained from roots, FH1:RH1—combination of essential oils obtained from flower heads and rhizomes in a ratio 1:1, FH1:RH3—combination of essential oils obtained from flower heads and rhizomes in a ratio 1:3, FH3:RH1—combination of essential oils obtained from flower heads and rhizomes in a ratio 3:1. Results of one-way ANOVA test, followed by a Tukey’s multiple comparison test, *n* = 3. Values designated by different small letters are significantly different (*p* < 0.05).

The results of the diffusion test in a solid medium and measurement of the zones of bacterial growth inhibition showed different reactions of the analyzed bacterial strains to the different EO types (Figure 1). The one-way ANOVA results showed a statistically significant effect of the different EO compositions on the bacterial growth inhibition in the case of EF (F = 5.95, *p* < 0.01), EFC (F = 6.43, *p* < 0.01), EC (F = 4.82, *p* < 0.05), PA (F = 10.71, *p* < 0.001), and CA (F = 7.58, *p* < 0.01). There was no effect of the different EO compositions on the growth zone in the case of SA (F = 1.31, *p* > 0.05) and SE (F = 1.81, *p* > 0.05). The largest zones (approx. 120 mm) of growth inhibition of SE, EFC, and CA were produced by RH, FH1:RH3, and RH, respectively. The difference in the bacterial reaction to the EO distilled from the flower heads, rhizomes, and roots was clearly visible in the case of PA. Namely, statistically significantly larger zones of growth inhibition were produced by FH than RH. In the case of EF, the zone of growth inhibition produced by FH3:RH1 was significantly lower compared to RO, FH1:RH1, and FH1:RH3. In the case of EFC and PA, the FH1:RH3 combination showed higher antibacterial activity than RH and RO and similar activity to that of FH. In turn, in the case of PA, the combination of EO distilled from the flower heads and rhizomes (FH1:RH1) resulted in the formation of a significantly larger zone of growth inhibition in relation to RH and a similar zone to that produced by FH. In contrast, in the case of CA, the growth inhibition zone produced by the combination of EOs distilled from the flower heads and rhizomes (FH1:RH1) was significantly lower than in the case of RH and RO but similar to the FH treatment.

**Figure 2:**
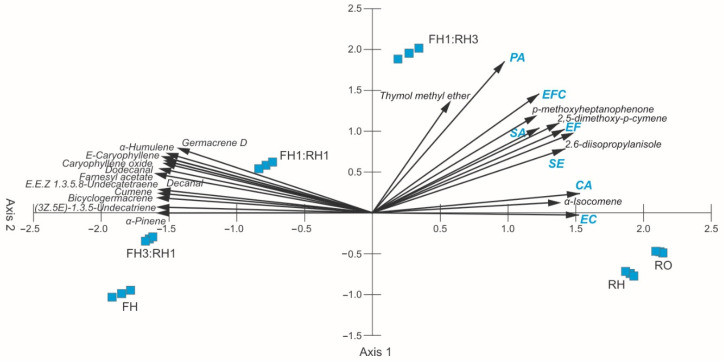
PCA ordination on the basis of the zones of bacterial growth inhibition induced by the *A. montana* essential oils and their combinations (blue squares). SA—*S. aureus* ATCC 25923, SE—*S. epidermidis* ATCC 12228, EF—*E. faecalis* PCM 896, EFC—*E. faecalis* (w.c.i.), EC—*E. coli* ATTC 25992, PA—*P. aeruginosa* ATCC 27853, CA—*C. acnes* ATCC 11827; FH—essential oil obtained from flower heads, RH—essential oil obtained from rhizomes, RO—essential oil obtained from roots, FH1:RH1—combination of essential oils obtained from flower heads and rhizomes in a ratio 1:1, FH1:RH3—combination of essential oils obtained from flower heads and rhizomes in a ratio 1:3, FH3:RH1—combination of essential oils obtained from flower heads and rhizomes in a ratio 3:1.

The results of the principal component analysis of the chemical composition of the essential oils and zones of bacterial growth inhibition are presented in Figure 2. The eigenvalues of the first (46.53) and second axis (18.74) indicated the presence of two gradients. The first two axes explained 91.9% of the variability (65.5%—Axis 1 and 26.4%—Axis 2). The RH and RO samples α-isocomene, 2,6-diisopropylanisole, 2,5-dimethoxy-p-cymene, and p-methoxyheptanophenone as the main components of EO distilled from the rhizomes and roots, and the ranges of zones of growth inhibition of EC, CA, SE, EF, SA, and EFC were positively correlated with Axis 1, whereas negative correlations were found for E-caryophyllene, germacrene D, and 10 essential oil components contained in EO distilled from the flower heads, whose share was over 2% (Table 1, Figure 2). Therefore, the growth inhibition zones of the bacterial strains were determined by these essential oil components. Two groups of samples can be distinguished in the ordination space (Figure 2, Table 1). The RH and RO samples are located on the right side. The close location of vectors representing the main components contained in EO obtained from the underground organs and the growth inhibition zones of the EC, CA, SE, EF, SA, and EFC bacteria proves the bactericidal effect of these metabolites (2,5-dimethoxy-p-cymene, 2,6-diisopropylanisole, p-methoxyheptanophenone) on the analyzed bacterial strains (Figure 2, Table 1). In turn, the main components of FH create the second group and are located in the left part of the ordination space. The negative correlation between E-caryophyllene, germacrene D, caryophyllene oxide, and the other components contained in the EO distilled from the flower heads and the growth inhibition zones of the bacterial strains show a significantly lesser role of these metabolites as antibacterial molecules (Figure 2).

The data obtained in the MIC test confirmed the earlier results obtained in the screening test. Namely, all the tested mountain arnica EOs exhibited a similar level of antibacterial activity and were characterized by the lowest (i.e., the most favorable) MIC values against *S. epidermidis* (from 50 µg/mL to 200 µg/mL) and against *S. aureus* (from 100 µg/mL to 400 µg/mL) (Table 2). The MIC against reference and hospital-associated enterococci of all the oils remained at the same beneficial level of 200 µg/mL. In contrast, the least favorable MIC values in the range from 200 µg/mL to 800 µg/mL were exhibited by the tested EOs against the Gram-negative *E. coli* and *P. aeruginosa* strains. Therefore, the spectrum of action of the tested mountain arnica EOs is directed more towards Gram-positive strains than Gram-negative ones, in both cases remaining at a moderate level.

The content and relationships of the main mountain arnica EO components, especially: E-caryophyllene, caryophyllene oxide, germacrene D, and 2,5-dimethoxy-p-cymene, are similar to the chemical EO profile in such species as *Origanum vulgare* [43], *Eupatorium intermedium* [44], *Centaurea intricata* [45], *Copaifera langsdorffii* [46], and *Eupatorium triplinerve* [47]. E-caryophyllene is a sesquiterpene compound exhibiting a wide anti-inflammatory [43], antioxidant, antibiotic [48], and antiparasitic activities [49] as well anticancer and analgesic properties [50]. Moghrovyan and Sahakyan [51] showed possible mechanisms of action of E-caryophyllene through disruption of the bacterial cell membrane. Caryophyllene oxide was found to exhibit a wide antioxidant, antiparasitic [52], and insecticidal activity [53]. Essential oils of many Verbenaceae family species are alternative sources of E-caryophyllene and germacrene D exhibiting antibacterial activity [54]. The therapeutic potential of 2,5-dimethoxy-p-cymene and thymol methyl ether, i.e., compounds isolated from *A. montana* essential oils, was presented by Jain et al. [55]. As presented above, the main components and their interactions were involved in their biological activity; however, understanding the efficacy and mechanism of action of each EO constituent is crucial for predicting the overall biological activity of EOs. This is very important in the context of our further studies of the antibacterial activity of *A. montana* essential oils.

### 2.4. Cytotoxic Activity

**Figure 3:**
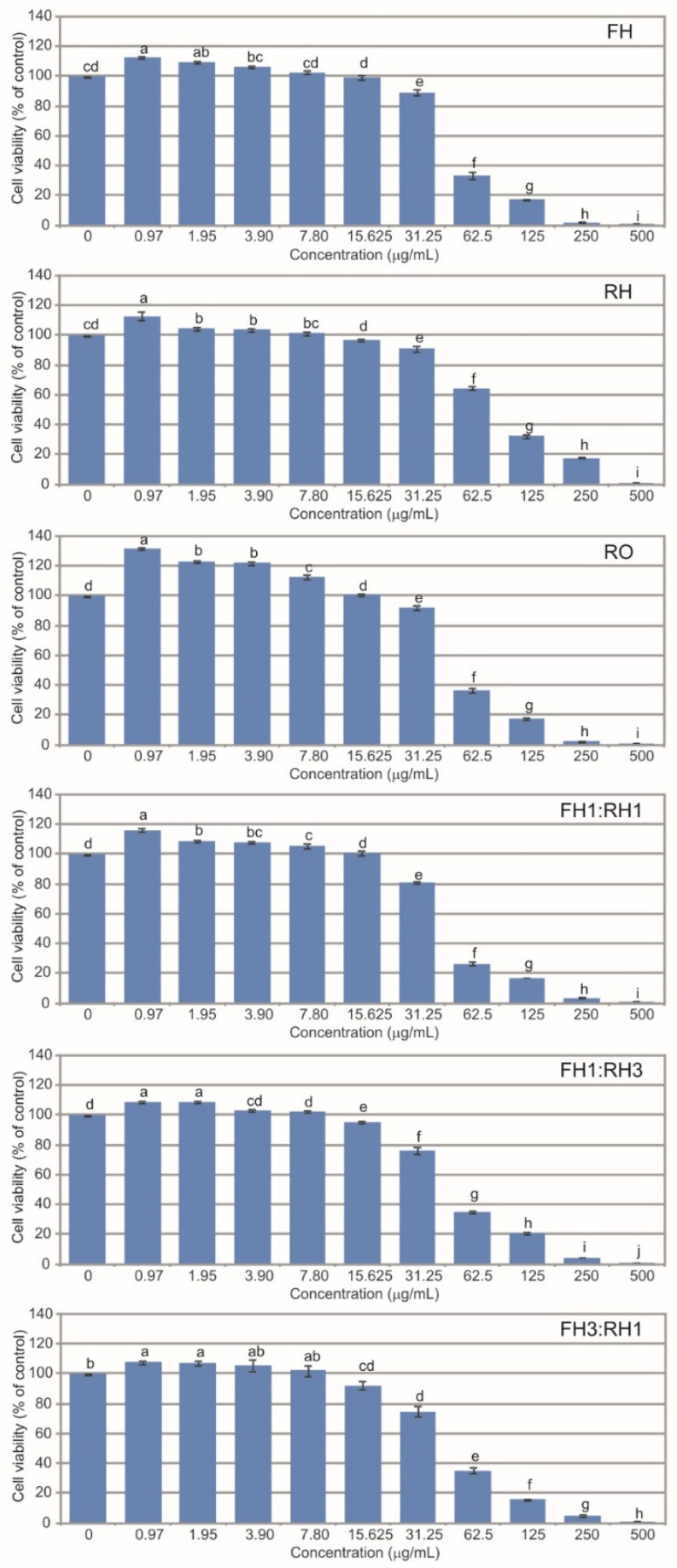
Cytotoxic activity of *A. montana* essential oils towards normal human skin fibroblasts. The values designated by different small letters are significantly different, Tukey test, *p* < 0.05. FH—essential oil obtained from flower heads, RH—essential oil obtained from rhizomes, RO—essential oil obtained from roots, FH1:RH1—combination of essential oils obtained from flower heads and rhizomes in a ratio 1:1, FH1:RH3—combination of essential oils obtained from flower heads and rhizomes in a ratio 1:3, FH3:RH1—combination of essential oils obtained from flower heads and rhizomes in a ratio 3:1. Results of one-way ANOVA test, followed by a Tukey’s multiple comparison test, *n* = 3. Values designated by different small letters are significantly different (*p* < 0.05).

All the *A. montana* essential oils exhibited concentration-dependent cytotoxicity toward normal human fibroblasts (BJ), with the highest concentrations tested (500–62.5 µg/mL) showing cytotoxic effects (Figure 3). At 31.25 µg/mL, FH, RH, RO, and FH1:RH1 caused a statistically significant reduction in fibroblast viability, compared to the control, but the values remained above the commonly accepted cytotoxicity threshold of 70%. FH3:RH1 showed a similar statistically significant reduction already at 15.625 µg/mL, whereas FH1:RH3 did not produce a significant change at this concentration. Moreover, all the analyzed EOs applied at the specific concentration ranges had a positive effect on the growth of the BJ cells, enhancing their viability. Interestingly, a 20–30% increase in cell viability, in relation to the control, was observed at the concentration of 0.97–3.90 µg/mL in the case of the essential oil obtained by distillation of the roots. In an earlier study, we showed that essential oils distilled from achenes, rhizomes, and roots had no effect on cell death initiation in normal fibroblasts [11,20]. The induction of apoptosis (but not autophagy or necrosis) at a level of 28.5–32.3% in human glioblastoma multiforme T98G and anaplastic astrocytoma MOGGCCM cell lines was a promising result in the context of anticancer activity [20]. Thus, this experiment showed that the toxicity of extracts derived from the mountain arnica essential oils obtained from underground plant parts is highly dependent on the concentration used, and their compounds seem to be the most promising and safe (in adequate solutions) groups of interesting molecules for further biomedical applications.

Preparations containing extracts from *A. montana* are widely used in cosmetic products for topical application (creams, ointments, gels), particularly for alleviating inflammation, swelling, and post-traumatic pain and supporting wound healing. Clinical studies have demonstrated that *A. montana* gel was as effective and well tolerated as ibuprofen in the treatment of hand osteoarthritis [56]. A systematic review confirmed the beneficial effects of arnica preparations in reducing pain and inflammatory symptoms associated with sports injuries and surgical interventions [57]. Furthermore, physicochemical studies have shown that sesquiterpene lactones, i.e., active constituents of arnica, are capable of penetrating through the epidermis and reaching the dermal layers [58].

Although ISO 10993-5 was originally developed for assessment of the biocompatibility of medical materials, it is also widely applied as a reference framework for plant extracts and bioactive substances in vitro. According to this standard, a test item is considered non-cytotoxic if cell viability remains ≥70%, compared to the untreated control. In cosmetic safety evaluation, according to the SCCS and OECD guidelines [59], a threshold of ≥80% viability is generally adopted for dermal applications. In our study, the fibroblast viability values remained within these ranges, confirming that the tested *A. montana* essential oils comply with the commonly accepted safety criteria and may be considered potentially safe for further cosmetic and pharmaceutical applications.

### 2.5. Interactions Between Arnica Montana Essential Oils, Their Combinations, and Antibiotics Against Selected Bacteria

The data clearly indicate that the presence of *A. montana* essential oils in the interactions with commercial antibiotics has a beneficial effect on their antibacterial activity (Table 3). Namely, the tested interactions of EO with antibiotics showed beneficial additive effect-synergism (7 combinations), partial synergism (21 combinations), or no effect-neither synergistic nor antagonistic activity (35). None of the combinations had an antagonistic effect on each other. Synergism occurred between FH1:RH3 or RH and metronidazole or amoxicillin against both strains of staphylococci and the reference enterococci. Partial synergism was also most often obtained in a similar interaction: FH1:RH3, RO, FH1:RH1 and RH with metronidazole and with amoxicillin. Such a beneficial additive effect of selected combinations is important in terms of application, as it may allow a reduction in the concentrations of antibiotics used, which meets the WHO warnings related to the threat of bacterial antibiotic resistance. The WHO warns that, if the procedures related to the overuse and inappropriate use of antibiotics are not changed, bacterial antibiotic resistance will further develop and there will be no solutions to currently treatable infectious diseases [60,61,62].

Reduced antibiotic effectiveness and antimicrobial resistance are one of the main global threats to public health [60,61,62]. Therefore, searching for alternative solutions is one of the key challenges of modern pharmacy and medicine [24]. The synergism between essential oil and antibiotics is a novel concept that has been recently reported by many researchers [63,64,65,66]. A study conducted by Drioiche et al. [63] revealed synergistic interactions between oregano essential oils and selected antibiotics. In the fight against resistant bacteria, *O. compactum* and *O. elongatum* essential oils proved promising when combined with antibiotics. Positive interactions between *Thymbra capitata* EO and antibiotics were observed by Maggio et al. [66]. As reported by Taibi et al. [30], the best synergistic action was demonstrated by a combination of *Ptychotis verticillata* EO with amoxicillin against *Micrococcus luteus* and a combination of this EO with ampicillin against *E. coli*. In turn, Zych et al. [64] found synergism between enrofloxacin and lavender EO more frequently than between enrofloxacin and cinnamon EO or clove EO.

Many reports have shown additivity or moderate synergism, indicating that EOs may offer possibilities for reducing antibiotic use, which addresses the WHO warnings related to the threat of bacterial antibiotic resistance [24,25,26,27,28,29]. This approach is one of the strategies for combating antibiotic-resistant bacteria and limiting the use of antibiotics and their release into the environment in the future [30,31,32]. The study presented in this paper clearly fits into this research trend. The synergy between mountain arnica essential oil obtained from different plant parts and antibiotics was analyzed for the first time. The results presented in Table 3 showed that both the combination of EO with antibiotics and the combination of the mixture of EO distilled from the flower heads and rhizomes with antibiotics had a very positive effect. However, the FICI values demonstrated different levels of interactions against the selected bacteria. The EO distilled from the rhizomes was shown to have the highest potential to be used in combination with antibiotics.

Numerous studies have investigated the synergistic interaction of antibiotics with various combinations of essential oil from medicinal plants. Karadağ et al. [67] reported a synergistic interaction between a combination of *Foeniculum vulgare* essential oil with amoxicillin against *E. faecalis* and *E. coli* pathogens. In turn, the results obtained by Soulaimani et al. [68] showed that individual EOs from *Ammodaucus leucotrichus* (dominated by L-perillaldehyde, D-limonene, and bornyl angelate), *Thymus vulgaris* (dominated by thymol, p-cymene, and g-terpinene), and *Lavandula maroccana* (dominated by carvacrol) and their optimal mixture revealed total synergism with this antibiotic against *S. aureus* and *P. aeruginosa*. In the present study, FH exhibited partial synergism with amoxicillin against *S. epidermidis* and *C. acnes*. Similarly, partial synergistic interaction between amoxicillin and RO against *S. aureus*, *S. epidermidis*, and *C. acnes* was found. Special attention should be paid to the interaction between metronidazole and RH, as the combination of metronidazole with RH exhibited synergistic interaction against *S. aureus* and *S. epidermidis* and partial synergism against *C. acnes*. Ibrahim et al. [69] underlined the role of extracts from such plants as *Symphytum officinale* and *Panax ginseng* against *Porphyromonas gingivalis* applied separately and in combination with metronidazole. The results reported by Piasecki et al. [70] showed additive or synergistic effects between *Mentha* spp. EO and metronidazole against reference and clinical *Helicobacter pylori* strains. The synergistic interactions in a combination of EO from medicinal plants with the common antibiotic ciprofloxacin have also been investigated. A combination of ciprofloxacin with *Thymus vulgaris* EO was found to enhance antibiotic activity and affect biofilm cell viability [71]. In turn, the antibacterial activity of EO from *Croton tetradenius* and its synergistic effect with ciprofloxacin was presented by Siqueira et al. [72], and a synergistic interaction between ciprofloxacin and *Rosmarinus officinalis* essential oil was reported by El Alama et al. [73]. The synergistic interaction between ciprofloxacin and FH against *C. acnes* was detected in the present study. Moreover, the partial synergistic interaction of RH and RO with amoxicillin and metronidazole against these bacterial strains suggests the need to continue the research on the use of RH and RO in combination with antibiotics to increase their effectiveness in combating other strains of microaerobic bacteria.

Investigations into the synergistic effects of mixtures of essential oils with conventional antibiotics have shown particularly promising results in inhibiting the growth of resistant strains [67,71,74,75,76,77]. Recent studies have demonstrated the significant therapeutic potential of essential oils in the fight against antibiotic-resistant bacteria [78,79]. The new research trend of using natural products involves optimization of the antibacterial effects of essential oils by improvement of EO mixture ratios for maximal antibacterial and antioxidant effectiveness [68,75]. The present results are the first step showing that different mixture ratios of two types of mountain arnica EO can have different antibacterial effectiveness (Table 3). The results showed that the FH1:RH3 combination (63 EO components with a 75% share of RH) revealed total synergism with amoxicillin and partial synergism with ciprofloxacin against *S. aureus*, whereas no effect was found between FH and the two antibiotics or between RH and these antibiotics. Similarly, the FH1:RH3 combination exhibited total synergism with metronidazole and partial synergism with amoxicillin against *E. faecalis*, whereas no effect was detected separately between FH and the two antibiotics or between RH and these antibiotics. In turn, the FH1:RH3 combination exhibited total synergism with amoxicillin against *S. epidermidis*, whereas no effect was found between FH and amoxicillin and partial synergism was noted between RH and amoxicillin against *S. epidermidis*. Probably, the presence of the greater number of metabolites and the new EO compositions resulted in different interactions between the EO components and between the EO components and the antibiotics. Therefore, in further research on the biological properties of arnica essential oils, attention should be paid to increasing their efficacy and effectiveness by optimizing blends.

## 3. Materials and Methods

### 3.1. Collection of Raw Material and Assay of the Essential Oil Content

The raw material was obtained from 4-year-old mountain arnica plants growing on grey-brown podzolic soil of an experimental field at the University of Life Sciences in Lublin, eastern Poland (51°31′39.5″ N, 22°45′6.7″ E). The flower heads were collected in the full flowering phase. This phase is characterized by the highest content of EO in the inflorescence [16]. In turn, the underground parts were obtained after collection of the flower heads. After harvesting, the flower heads, rhizomes, and roots were transferred to the laboratory and air-dried at 25 °C. The raw material was dried, prepared, and distilled according to procedures described previously [6,20]. The essential oils were collected over water, separated, dried over anhydrous sodium sulfate, and stored in the dark at 4 °C prior to GC-MS analysis. The analysis was carried out in four repetitions.

### 3.2. GC-MS Analysis

The chromatographic separation of volatile constituents was carried out using a Varian 4000 GC–MS/MS instrument (Varian, Palo Alto, CA, USA) equipped with a VF-5ms capillary column (30 m × 0.25 mm × 0.25 μm). The oven temperature program was initiated at 50 °C and raised to 250 °C at 4 °C/min. The injector temperature was set at 250 °C, the injection volume was 5 μL, and the split ratio was 1:50. Mass spectra were recorded in an electron impact (EI) mode at 70 eV, with an ion source temperature of 200 °C over the range of 40–870 *m*/*z*. Each type of essential oil was analyzed in triplicate, following procedures described previously [6,16].

### 3.3. Qualitative and Quantitative Analysis

Compounds were identified based on complementary criteria:

Retention indices (RI) were calculated by the van den Dool–Kratz equation relative to a homologous series of n-alkanes (C_8_–C_28_) analyzed in identical chromatographic conditions. The experimental RI values were compared with literature data for nonpolar stationary phases [34,80] and with NIST retention index databases [81].

Mass spectral matching was performed against the NIST EI-MS library [82]. A compound was considered reliably identified when its experimental RI differed by no more than ±10–15 units from the reference values and the spectral similarity index exceeded ~85–90%.

Manual verification was additionally performed by visual inspection of diagnostic ions and fragmentation patterns, in line with standard recommendations for essential oil analysis [83] and more recent guidance on mass spectral library searches [80,81].

Compounds that met both the RI and MS criteria were classified as identified, while those fulfilling only one condition were regarded as tentatively identified. Relative abundances were calculated from GC peak areas without response factor correction. For calibration of the RI values and quantification of the relative composition, n-alkanes C_12_ and C_19_ were used as internal standards in accordance with previously published procedures [84].

The following EOs were used in all the experiments: essential oil obtained from the flower heads (FH), essential oil obtained from the rhizomes (RH), essential oil obtained from the roots (RO), a combination of essential oils obtained from the flower heads and rhizomes in a ratio 1:1 FH1:RH1, a combination of essential oils obtained from the flower heads and rhizomes in a ratio 1:3 (FH1:RH3), and a combination of essential oils obtained from the flower heads and rhizomes in a ratio 3:1 (FH3:RH1).

### 3.4. Antibacterial Activity

#### 3.4.1. Microorganisms and Materials

The study was conducted using the following aerobic strains: Gram-positive: *Staphylococcus aureus* ATCC 25923, *Staphylococcus epidermidis* ATCC 12228, *Enterococcus faecalis* PCM 896, and *Enterococcus faecalis* (wound culture infection—w.c.i.) clinical isolates from the Gastroenterology Department, Gram-negative *Escherichia coli* ATCC 25992 and *Pseudomonas aeruginosa* ATCC 278532, and *Cutibacterium acnes* ATCC 11827. All strains are properly stored at the Department of Biochemistry and Biotechnology of the Medical University of Lublin, Poland.

The following microbiological tests were used: Müeller-Hinton (M-H), agar, Oxoid Ltd. (Basingstoke, UK), and broth, Oxoid Ltd. (Basingstoke, UK).

All the antimicrobial assays were performed in aseptic conditions and in three independent experiments, each conducted in triplicate, and yielded consistent results across all the replicates.

#### 3.4.2. Screening of Plant Essential Oils for Antibacterial Activity-Agar Diffusion Assay

For the preliminary evaluation, essential oil solutions were prepared in DMSO at a concentration of 10 mg/mL. Mueller-Hinton agar was poured into Petri dishes according to the manufacturer’s protocol [85]. A bacterial suspension equivalent to 0.5 McFarland standard (1.5 × 10^8^ CFU/mL) was prepared and spread evenly on the agar surface with a sterile swab. The designated areas of the plates were exposed to 10 µL of arnica essential oil samples (corresponding to 100 µg). The plates were then incubated in aerobic conditions at 37 °C for 24 h. Following the incubation, the diameters of inhibition zones surrounding the oil samples were determined using a microbiological ruler.

#### 3.4.3. Determination of the Minimum Inhibitory Concentration (MIC) of Essential Oils

The MIC, defined as the lowest concentration of a compound that prevents visible microbial growth, was determined using the standard broth microdilution method in 96-well microplates. Essential oils were prepared as serial dilutions in broth, ranging from 1600 µg/mL to 6.25 µg/mL. Each well was inoculated with 2 µL of the bacterial suspension adjusted to 0.5 McFarland. The assay included positive controls to confirm bacterial viability, negative controls to ensure sterility, and reagent controls to account for the intrinsic color of the essential oils across the dilutions. The plates were incubated in appropriate conditions, and the MIC values were recorded based on the absence of visible growth.

#### 3.4.4. Synergistic Interactions Between EO and Antibiotics

The synergistic effects of arnica oils and commercial antibiotics were evaluated using the checkerboard method based on determination of the Minimum Inhibitory Concentration (MIC). Specifically, amoxicillin (Polfa Tarchomin, Warszawa, Poland), metronidazol, and ciprofloxacin (both from Polpharma S.A., Starogard Gdański, Poland) were tested. Stock solutions and serial twofold dilutions of the antibiotics as well as dilutions of oil samples corresponding to the fourfold MIC value were prepared. In total, 50 μL of broth was distributed into each well of the microdilution plates. The first tested antibiotic of the combination was serially diluted vertically, while the arnica oil sample was diluted horizontally in the 96-well plate. Following this, 100 μL of bacterial inoculum at a concentration of 1.5 × 10^8^ CFU/mL was added to each well, and the plates were incubated in suitable conditions. The resulting checkerboard layout included each combination of the tested agents as well as determination of the MIC for each agent used individually. The fractional inhibitory concentration index (FICI) was calculated for each combination of the concentrations of the agents using the following formula: FICI = (MIC_A/B_/MIC_A_) + (MIC_B/A_/MIC_B_), where MIC_A_ is the MIC of agent A alone, MIC_A/B_ is the MIC of agent A in combination with agent B and MIC_B_, and MIC_B/A_ is defined analogously as agent A. The obtained FICI values indicate total synergism (FICI ≤ 0.5), partial synergism (0.5 < FICI ≤ 0.75), no effect (0.75 < FICI ≤ 2), or antagonism (FICI > 2) between two agents.

### 3.5. Cytotoxic Activity

#### 3.5.1. Cell Culture Experiments

Cytotoxicity tests were conducted using the normal human fibroblast cell line BJ (CRL-2522, ATCC-LGC Standards, Teddington, UK). The cells were cultured in Eagle’s Minimum Essential Medium (American Type Culture Collection-LGC, Teddington, UK) supplemented with 10% Fetal Bovine Serum (Pan-Biotech Gmbh, Aidenbach, Germany), 100 U/mL penicillin, and 0.1 mg/mL streptomycin (Sigma-Aldrich, St. Louis, MO, USA). The cultures were maintained at 37 °C in a humidified atmosphere containing 5% CO_2_ (Heraeus Cytoperm 2, Thermo Scientific, Waltham, MA, USA).

#### 3.5.2. Cell Viability

Essential oils were initially dissolved in DMSO to obtain a stock solution at a concentration of 100 mg/mL. Subsequently, serial dilutions of the oils were prepared in the EMEM culture medium with 2% FBS to yield final concentrations ranging from 0.97 to 500 µg/mL. Human fibroblasts (BJ) were seeded into 96-well plates at a density of 1 × 10^4^ cells per well in 100 µL of culture medium. After 24 h of incubation at 37 °C in a humidified atmosphere containing 5% CO_2_, the culture medium was replaced with 100 µL of the tested compounds at the specified concentrations (*n* = 3). In parallel, DMSO controls were included at all corresponding concentrations to exclude any potential solvent-induced cytotoxicity. Following 24 h exposure to the tested compounds, cell viability was assessed using the MTT assay (3-(4,5-dimethylthiazol-2-yl)-2,5-diphenyltetrazolium bromide (Sigma-Aldrich, St. Louis, MO, USA). The compounds and DMSO controls were removed, and 100 µL of culture medium containing 1 mg/mL MTT was added to each well. After 3-h incubation, 100 µL of the SDS (Sigma-Aldrich, St. Louis, MO, USA) solution prepared in 0.01 M HCl (Avantor Performance Materials Poland S.A., Gliwice, Poland) was added to each well. The plates were then incubated for another 12 h, after which absorbance was measured at 570 nm using a microplate reader (BioTek Synergy, Vinooski, VA, USA). The results were expressed as a percentage of absorbance relative to the negative cytotoxicity control (cells cultured in medium without essential oils), which was considered to represent 100% viability.

### 3.6. Statistical Analysis

The statistical analyses were carried out using the Statistica 6.0 software (Stat. Soft, Inc., Krakow, Poland). One-way ANOVA and subsequent Tukey’s tests were used. The differences were considered significant at *p* < 0.05. The principal component analysis (PCA) was applied to explain the relationships between the components of the different essential oil types obtained by hydrodistillation and the zones of bacterial growth inhibition induced by the mountain arnica EOs and their combinations. Prior to the PCA, the data were centered and log-transformed. The analyses were carried out using the statistical package (MVSP) program version 3.1 [86].

## 4. Conclusions

Individual plant parts, namely flower heads (*Arnicae anthodium*), rhizomes (*Arnicae rhizome*), and roots (*Arnicae radix*) can be determinants of the essential oil composition. For the first time, we demonstrate the significant variation in the composition of EO from different plant organs of the same plants and, in consequence, their different antibacterial activity. E-caryophyllene, caryophyllene oxide, germacrene D, farnesyl acetate, dodecanal, and decanal were the main ingredients in the essential oil of the flower heads. The last three molecules mentioned were not found in the underground part. In turn, 2,5-dimethoxy-p-cymene, 2,6-diisopropylanisole, p-methoxyheptanophenone, thymol methyl ether, and α-isocomene were the main components of the essential oils from the rhizomes and roots. Such a wide variety of EO compositions proves their antibacterial activity and high functionality in terms of the use of secondary metabolites of this plant species. Moreover, the synergy between different types of mountain arnica essential oils and their mixtures and antibiotics against different bacterial strains was analyzed for the first time. The data clearly indicate that the presence of arnica EO in interaction with commercial antibiotics (amoxicillin, ciprofloxacin, metronidazole) has a beneficial effect on their antibacterial activity. The findings from this study highlight the necessity for additional research into the biological activity of mountain arnica essential oils (both alone and in combination with antibiotics) against other bacterial strains, the use of essential oils as an experimental factor in in vivo studies, and further exploration of their mechanisms of action.

## Figures and Tables

**Table 1:** Composition of essential oils obtained from *A. montana* flower heads (FH), rhizomes (RH), and roots (RO).

Compounds	IR	FH		RH		RO	
		[%]	±SD	[%]	±SD	[%]	±SD
Cumene	933	4.21	±0.173	-	-	-	-
α-Pinene	940	2.02	±0.063	-	-	-	-
Camphene	956	0.52	±0.013	-	-	-	-
Thuja-2.4(10)-diene	961	0.51	±0.012	-	-	-	-
β-Pinene	983	0.39	±0.011	-	-	-	-
dehydro-1.8-Cineole	995	0.69	±0.021	-	-	-	-
n-Octanal	1002	0.51	±0.013	-	-	-	-
α-Phellandrene	1012	0.85	±0.025	-	-	-	-
p-Cymene	1029	0.66	±0.017	-	-	-	-
Limonene	1032	0.56	±0.013	0.08	±0.002	-	-
Linalool	1106	0.81	±0.023	-	-	-	-
Nonanal	1113	1.87	±0.056	-	-	-	-
cis-p-Menth-2-en-1-ol	1130	0.13	±0.003	-	-	-	-
(3Z.5E)-1.3.5-Undecatriene	1180	2.17	±0.069	-	-	-	-
E.E.Z 1.3.5.8-Undecatetraene	1185	2.18	±0.074	-	-	-	-
γ-Terpineol	1198	-	-	0.18	±0.004	0.20	±0.003
Decanal	1215	5.46	±0.202	-	-	-	-
trans-Carveol	1220	-	-	0.40	±0.018	0.42	±0.006
Thymol methyl ether	1238	2.45	±0.093	6.53	±0.230	1.45	±0.001
Carvacrol methyl ether	1249	0.57	±0.015	0.17	±0.005	-	-
Thymol	1304	1.12	±0.037	0.41	±0.008	0.46	±0.002
Carvacrol	1313	2.00	±0.074	1.53	±0.066	1.56	±0.058
Silphiperfol-5-ene	1326	-	-	0.11	±0.003	0.23	±0.003
Presilphiperphol-7-ene	1345	-	-	0.07	±0.002	0.15	±0.002
7-epi-silphiperfol-5-ene	1348	0.48	±0.018	0.24	±0.010	0.41	±0.003
α-Longipinene	1354	0.16	±0.004	-	-	-	-
Silphiperfol-6-ene	1375	-	-	0.07	±0.000	0.13	±0.003
Eugenol	1383	1.64	±0.052	-	-	-	-
Modheph-2-ene	1384	-	-	0.08	±0.003	0.14	±0.002
β-Maaliene	1385	0.32	±0.008	-	-	-	-
α-Isocomene	1394	1.65	±0.056	0.99	±0.009	2.23	±0.027
Longifolene	1416	0.12	±0.003	-	-	-	-
2,5-dimethoxy-p-cymene	1417	-	-	49.84	±1.679	52.21	±0.656
Dodecanal	1419	6.24	±0.212	-	-	-	-
E-Caryophyllene	1427	15.84	±0.649	0.91	±0.010	0.36	±0.014
α-trans-Bergamothene	1439	1.08	±0.033	0.44	±0.000	0.47	±0.018
2.6-diisopropylanisole	1439	-	-	19.30	±0.396	20.76	±0.108
(Z)β-Farnesene	1445	1.29	±0.036	-	-	-	-
(E)β-Farnesene	1459	1.06	±0.028	-	-	-	-
α-Humulene	1467	2.18	±0.070	0.09	±0.000	0.08	±0.001
p-methoxyheptanophenone	1478	-	-	14.64	±0.351	12.43	±0.170
Germacrene D	1483	6.87	±0.257	0.22	±0.017	-	-
α-Amorphene	1487	1.36	±0.041	-	-	-	-
methyl-γ-Ionone	1489	0.21	±0.005	-	-	-	-
Bicyclogermacrene	1518	2.62	±0.076	-	-	-	-
Isobornyl isovalerate	1519	-	-	0.17	±0.004	0.19	0.002
δ-Amorphene	1529	0.13	±0.004	-	-	-	-
Zonarene	1531	0.60	±0.014	-	-	-	-
β-sesquiphellandrene	1535	0.09	±0.002	0.28	±0.003	0.56	0.006
Lippifoli-1(6)-en-5-one	1567	0.58	±0.016	0.55	±0.008	1.82	0.025
Caryophyllene alcohol	1588	0.75	±0.022	-	-	-	-
Caryophyllene oxide	1593	7.86	±0.195	0.22	±0.021	0.45	0.022
n-Heksadecane	1600	0.97	±0.026	-	-	-	-
Humulene epoxide II	1623	0.50	±0.014	-	-	-	-
epi-α-Muurolol	1658	0.38	±0.010	-	-	-	-
Z-Amyl cinnamaldehyde	1661	0.16	±0.004	-	-	-	-
α-Cadinol	1670	1.84	±0.064	-	-	-	-
14-hydroxy-9-epi-E-Caryophyllene	1676	0.31	±0.009	-	-	-	-
Cadalene	1688	0.24	±0.007	-	-	-	-
Valeranone	1692	0.85	±0.025	-	-	-	-
n-heptadecane	1700	1.86	±0.067	-	-	-	-
Pentadecanal	1728	0.89	±0.025	-	-	-	-
Farnesyl acetate	1844	6.68	±0.265	-	-	-	
Monoterpene hydrocarbons		10.41		0.49		0.49	
Oxygenated monoterpenes		7.28		8.98		8.98	
Sesquiterpene hydrocarbons		55.43		4.27		4.27	
Oxygenated sesquiterpenes		2.22		0.00		0.00	
Aliphatic hydrocarbons		7.18		0.00		0.00	
Oxygenated aliphatic hydrocarbons		14.97		0.00		0.00	
Aromatic phenyl compounds and ketones		0.00		83.77		83.00	
Summary		97.49		97.51		96.74	

RI—retention indices from temperature-programming following the definition proposed by Van Den Dool and Kratz [34].

**Table 2:** Minimum inhibitory concentration (MIC) and minimum bactericidal concentration of *A. montana* essential oils. SA—*S. aureus* ATCC 25923, SE—*S. epidermidis* ATCC 12228, EF—*E. faecalis* PCM 896, EFC—*E. faecalis* (w.c.i.), EC—*E. coli* ATTC 25992, PA—*P. aeruginosa* ATCC 27853, CA—*C. acnes* ATCC 11827; FH—essential oil obtained from flower heads, RH—essential oil obtained from rhizomes, RO—essential oil obtained from roots, FH1:RH1—combination of essential oils obtained from flower heads and rhizomes in a ratio 1:1, FH1:RH3—combination of essential oils obtained from flower heads and rhizomes in a ratio 1:3, FH3:RH1—combination of essential oils obtained from flower heads and rhizomes in a ratio 3:1.

	SA	SE	EF	EFC	EC	PA	CA
FH	200	200	200	200	400	400	200
RH	100	50	200	200	200	400	100
RO	200	200	200	200	200	800	200
FH1:RH1	200	100	200	200	400	800	400
FH1:RH3	200	100	200	200	200	800	200
FH3:RH1	400	200	200	200	400	800	200

**Table 3:** Interactions between *A. montana* essential oils, their combinations, and antibiotics tested against selected bacteria. FH—essential oil obtained from flower heads, RH—essential oil obtained from rhizomes, RO—essential oil obtained from roots, FH1:RH1—combination of essential oils obtained from flower heads and rhizomes in a ratio 1:1, FH1:RH3—combination of essential oils obtained from flower heads and rhizomes in a ratio 1:3, FH3:RH1—combination of essential oils obtained from flower heads and rhizomes in a ratio 3:1.

Bacteria	Antibiotic	FH	RH	RO	FH1:RH1	FH1:RH3	FH3:RH1
	Amoxicillin	1.5^n^	1.06^n^	0.562^p^	1.06^n^	0.5^s^	1.5^n^
*S. aureus* ATCC	Ciprofloxacin	1.06^n^	1.06^n^	1.06^n^	1.06^n^	0.75^p^	1.06^n^
25923	Metronidazole	1.06^n^	0.375^s^	0.75^p^	1.06^n^	0.5^s^	1.06^n^
	Amoxicillin	1.06^n^	0.562^p^	0.562^p^	0.562^p^	0.5^s^	0.562^p^
*S. epidermidis* ATCC	Ciprofloxacin	1.5^n^	0.75^p^	1.5^n^	1.5^n^	0.75^p^	1.06^n^
12228	Metronidazole	1.06^n^	0.375^s^	0.75^p^	0.75^p^	0.5^s^	1.06^n^
	Amoxicillin	1.5^n^	1.06^n^	1.06^n^	1.06^n^	0.75^p^	1.06^n^
*E. faecalis* PCM 896	Ciprofloxacin	1.5^n^	1.5^n^	1.5^n^	1.5^n^	1.5^n^	1.5^n^
	Metronidazole	1.06^n^	1.5^n^	0.562^p^	1.06^n^	0.5^s^	1.5^n^
	Amoxicillin	1.5^n^	0.75^p^	0.562^p^	1.25^n^	1.25^n^	1.5^n^
*C. acnes* ATCC 11827	Ciprofloxacin	1.06^n^	0.375^s^	1.25^n^	0.562^p^	0.75^p^	1.06^n^
	Metronidazole	1.5^n^	0.75^p^	0.562^p^	1.5^n^	1.06^n^	1.06^n^

Fractional inhibitory concentration index; ^s^Total synergism; ^p^Partial synergism; ^n^No effect; total synergism (FICI ≤ 0.5), partial synergism (0.5 < FICI ≤ 0.75), no effect (0.75 < FICI ≤ 2) between two agents.

## Data Availability

The raw data supporting the conclusions of this article will be made available by the authors on request. Currently, the basic data is deposited with several co-authors and will be made available to the publisher if necessary.
